# Sexual aspects of liver transplant candidates and recipients: evidence available in the literature^1^

**DOI:** 10.1590/1518-8345.2744.3033

**Published:** 2018-09-03

**Authors:** Jennifer Tatisa Jubileu Magro, Karina Dal Sasso Mendes, Cristina Maria Galvão

**Affiliations:** 2 MSc, RN, Hospital das Clínicas, Faculdade de Medicina de Ribeirão Preto, Universidade de São Paulo, Ribeirão Preto, SP, Brazil.; 3 PhD, RN, Escola de Enfermagem de Ribeirão Preto, Universidade de São Paulo, PAHO/WHO Collaborating Centre for Nursing Research Development, Ribeirão Preto, SP, Brazil.; 4 PhD, Full Professor, Escola de Enfermagem de Ribeirão Preto, Universidade de São Paulo, PAHO/WHO Collaborating Centre for Nursing Research Development, Ribeirão Preto, SP, Brazil.

**Keywords:** Perioperative Nursing, Sexuality, Liver Transplantation, Review, Health Services, Transplant Recipients

## Abstract

**Objective:**

to analyze the evidence available in the literature on the alterations in the sexuality of candidates and recipients of liver transplantation.

**Method:**

integrative review of the literature with search for primary studies in the databases MEDLINE (via PUBMED), CINAHL e LILACS, published in English, Portuguese and Spanish.

**Results:**

the 16 primary studies included were grouped into three categories: 1) female sexuality (n=5), 2) male sexuality (n=5) and 3) male and female sexuality (n=6). In category 1, the subjects investigated were contraception, pregnancy, sexual dysfunction, presence of gynecological symptoms and sexually transmitted infections. In category 2, the main focus of the studies was erectile dysfunction, sexual desire and satisfaction, and consequences of the immunosuppressive regimen with mycophenolic acid in men. In category 3, the evaluation of sexual function was the main topic.

**Conclusion:**

the scientific evidence generated provides support to encourage health professionals to incorporate the topic of sexuality in the routine of care. Knowledge gaps were identified and new studies should be conducted in order to implement interventions to prevent, minimize and/or control changes related to the patient’s sexuality.

## Introduction

End-stage liver disease significantly reduces quality of life. In this context, the transplantation can reverse the terminal stage and improve the patients’ health conditions^1^.

According to the World Health Organization (WHO), sexuality is a central aspect of being human throughout the life cycle that encompasses sex, gender identities, sexual orientation, eroticism, pleasure, intimacy and reproduction. It is manifested in different forms by the individuals and can be influenced by the interaction of biological, psychological, social, economic, political, cultural, historical and religious factors^2^. In general, there is evidence in the literature that both liver transplant candidates and recipients may suffer some degree of sexual dysfunction, either temporary or permanent^3^.

In liver transplant candidates, sexual dysfunction is common and related to the changes triggered by the chronic liver disease, to the drug therapy, hormonal changes, irrigation of the pelvis and emotional changes. Men may experience symptoms such as difficulty in erection, loss of libido, premature ejaculation and oligospermia. Women may be affected by ovarian dysfunction, amenorrhea, decreased or absent libido and infertility. Sexual health is complex because it involves different factors, such as age, use of medications, and social and psychological aspects coming from previous experiences or from the transplantation^4^ ^-^ ^5^.

The feelings of well-being and improved clinical status after transplantation are related to the success of the procedure. However, problems such as the side effects of immunosuppressive drugs and the risk of postoperative complications, such as the possibility of graft failure, rejections and vascular thrombosis, may be stressors for the patient^6^. Liver transplantation can improve the patients’ quality of life; however, there is evidence that not all patients experience the same benefits^7^.

The chronic condition of the patient in both phases of transplantation (before and after surgery) represents a battle for quality of life, which is altered by the disease and by the treatment it requires^7^. Thus, one of the first aspects of life affected by the physical and emotional symptoms is sexual function^8^. With the prospect of performing the transplant, the patient focuses his thoughts on survival and recovery. After the transplant, new perspectives and life plans emerge, allowing the resumption of intimacy and favoring sexual activity^4^.

Sexuality is a complex area of ​​human behavior and it should not be underestimated, both for the transplant candidate and the transplant recipient. Sexual function is influenced by disease, psychological distress, and imbalance of interpersonal relationships. In most cases, chronic disease is associated with sexual dysfunction and results in decreased sexual activity due to discomfort, fatigue and changes in body image, which significantly affects the patient’s relationship with the partner^7^ ^,^ ^9^.

A systematic review with meta-analysis of observational studies aimed to evaluate the effect of liver transplantation on endocrine and sexual function in adult patients. The results suggested that liver transplantation improves the hormonal perturbations associated with chronic liver disease by restoring physiological levels of growth hormone, insulin-like growth factor, testosterone, estradiol, prolactin, follicle-stimulating hormone and luteinizing hormone. The 21 studies analyzed demonstrated that liver transplantation was associated with improved sexual function^8^.

Given the above and considering that the subject of sexuality of candidates and recipients of solid organs is scarcely investigated, specifically regarding liver transplantation, and that health professionals need to be prepared to address this issue with patients in health services, the interest in conducting this integrative review is justified, since its results can generate evidence to support the care provided. Thus, the objective of the present review was to analyze the evidence available in the literature on the changes in the sexuality of candidates and recipients of liver transplantation.

## Method

Integrative review (IR) of the literature was the method of knowledge synthesis used. For this method, five steps were taken: elaboration of the research question (identification of the problem), literature search for primary studies, evaluation of primary studies, data analysis and presentation of the review^10^.

To guide the IR, based on the PECOT strategy (acronym for patient, exposure, comparison, outcomes and time), the following question was formulated: “What evidence is available in the literature about changes in the sexuality of liver transplant candidates and recipients?”. The first element (P = patient or problem) are the candidates and recipients of liver transplantation, the second (E = exposure) is sexuality, the third element (O = outcomes or results) are alterations related to sexuality and (T=time) is the pre- and post-operative periods.

The search for primary studies was performed through an online search in three health databases: *Medical Literature Analysis and Retrieval System Online* (MEDLINE via PUBMED), *Cumulative Index to Nursing and Allied Health Literature* (CINAHL) and Latin- American and Caribbean Health Sciences (LILACS).

**Figure 1:**
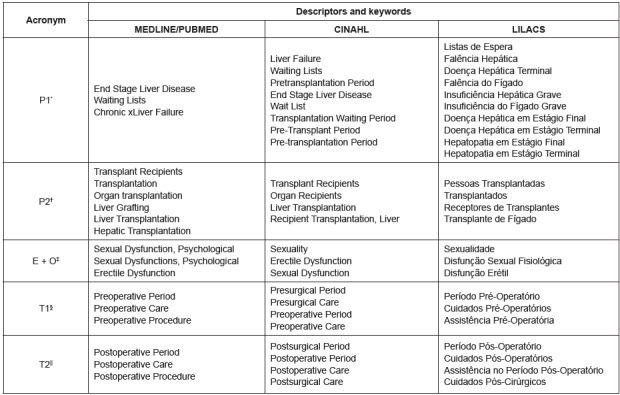
Descriptors and keywords selected in each database according to the acronym PECOT

The descriptors and keywords were established according to each database in order to guarantee a rigorous and broad search for the primary studies on the topic of interest, and were associated with the PECOT acronym (Figure 1). In order to search the publications in each database, the descriptors and the keywords were crossed with each other through tactical operations, using the Boolean operators AND and OR.

*P1 = patient crossing 1; †P2 = patient crossing 2; ‡E+O = exposure crossing + outcomes; §T1 = time crossing 1; ||T2 = time crossing 2

For the selection of the primary studies of this review, the following inclusion criteria were adopted: primary studies whose authors investigated the changes in the sexuality of candidates or recipients of liver transplantation, which were published in English, Portuguese and Spanish in the last 10 years (from June 30^th^, 2006 to June 30^th^, 2016). The time period was established to ensure an adequate number of studies, considering that a large number of primary studies could make it impossible to conduct the integrative review or introduce biases in the next steps of the method.

**Figure 2:**
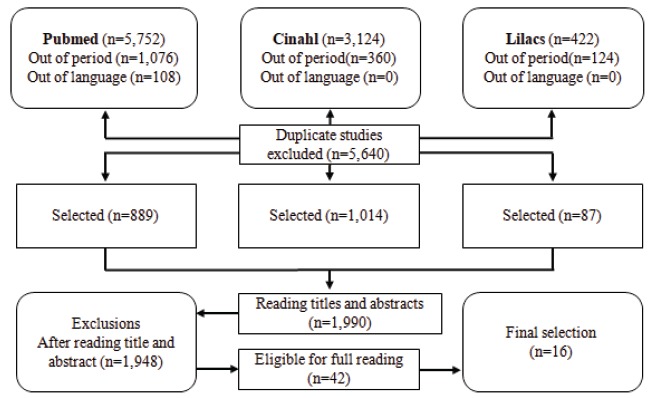
Flowchart for the selection of primary studies

From the results of the search strategies used in the databases selected for the review, a file was imported into the EndNote reference manager, version X5. For the management of references, folders were created for each database and duplicate studies, books, dissertations, thesis and other non-scientific texts, studies written in languages other than the established ones or published outside the established period were filtered. The pre-selection of the primary studies through the reading of the titles and abstracts was performed and, finally, the final selection of the studies for reading in full. Figure 2 shows the flowchart for the selection of primary studies.

An instrument developed by a national researcher was used to extract data from primary studies. The instrument contains a set of items that enable the identification of researches, methodological characteristics, main results and conclusions^11^. For the evaluation of the studies, the definition of the type of research described by the authors was maintained and, when this information was absent, the concepts described by nursing researchers in scientific methodology were used to classify the methodological approach used in the research^12^.

The hierarchy of evidence was classified according to the type of clinical question of the studies. The clinical question may be: (a) of significance (with five levels of evidence, the strongest being level I, evidence obtained from the meta-synthesis of qualitative studies, and the lowest, level V, evidence from expert opinion); (b) of prognosis, prediction or etiology (with five levels of evidence, the strongest being level I, evidence obtained from the synthesis of cohort or case-control studies, and the lowest, level V, evidence from expert opinion specialists) and (c) of intervention, treatment or diagnosis/diagnostic test (with seven levels of evidence the strongest being level I, evidence obtained from systematic review or meta-analysis, and the lowest, level VII, evidence from expert opinion)^13^.

The synthesis of the results of the review was descriptive. Thus, the 16 primary studies included in the review sample were grouped into three categories: (1) “female sexuality” (n=5), (2) “male sexuality” (n=5), and (3) “male and female sexuality” (n=6).

## Results

Regarding the database, 13 primary studies (81.2%) were identified in Medline (via PubMed) and three (18.7%) in CINAHL. Regarding the country of origin, six studies (37.5%) were from the United States of America, three (18.75%) from Poland, three (18.75%) from France and one (6.25%) from Canada, Switzerland, China and Taiwan, respectively. It was observed that 87.5% (n=14) of the primary studies were published in medical journals. Regarding the period of publication, one study (6.25%) was published in 2001, three (18.75%) in 2006, one (6.25%) in 2008, two (12.5%) in 2009, four (25%) in 2013, four (25%) in 2014 and one (6.25%) in 2015.

**Figure 3:**
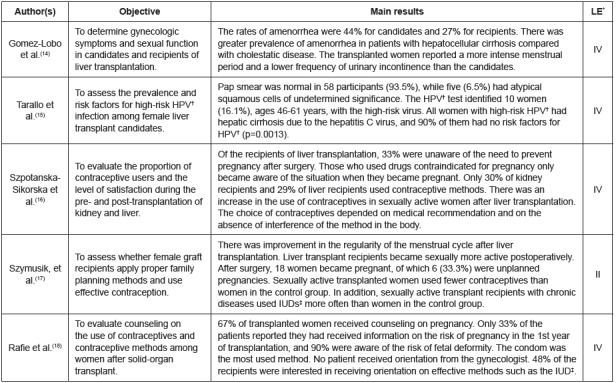
Characteristics of the primary studies included in the category “female sexuality”. Ribeirão Preto, SP, Brazil, 2017

**Figure 4:**
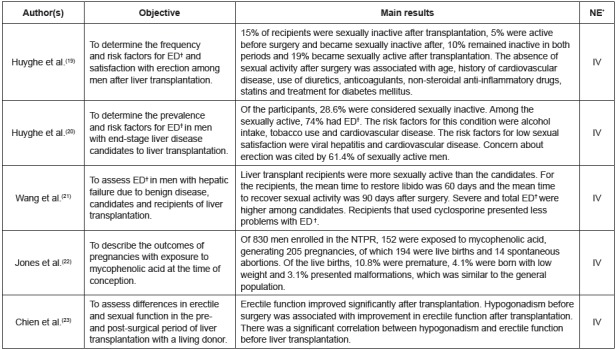
Characteristics of the primary studies included in the category “male sexuality”. Ribeirão Preto, SP, Brazil, 2017

**Figure 5:**
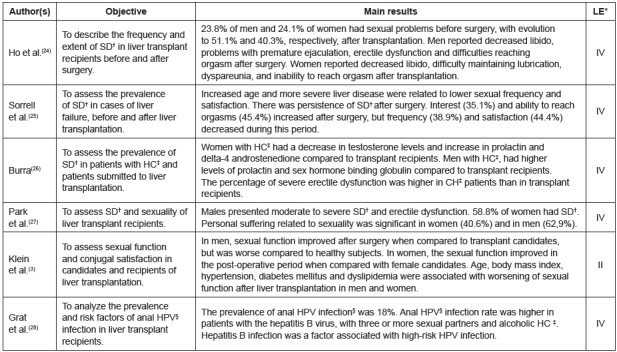
Characteristics of the primary studies included in the category “male and female sexuality”. Ribeirão Preto, SP, Brazil, 2017

The type of clinical question of all the primary studies included was Prognosis/Prediction or Etiology. Fourteen investigations (87.5%) were classified as level of evidence IV (evidence from a single qualitative or descriptive study) and two (12.5%) were classified as Level of Evidence II (evidence from a single cohort or case-control study). In Figures 3, 4 and 5, the knowledge synthesis of the primary studies included in the review is presented, according to each category.

*LE=level of evidence; ^†^HPV= human papillomavirus infection; ^‡^IUD= intrauterine device

*LE= level of evidence; ^†^ED=erectile dysfunction; ^‡^NTPR=*National Transplant Pregnancy Registry*

*LE=level of evidence; ^†^DS=sexual dysfunction; ^‡^HC= hepatic cirrhosis; ^§^HPV= human papillomavirus infection

## Discussion

Primary studies grouped in the category “female sexuality” had as main topics of investigation: contraception and pregnancy^16^ ^-^ ^18^, sexual dysfunction and of gynecological symptoms^14^, and sexually transmitted infections^15^. In women, hepatic cirrhosis can lead to the development of amenorrhea, premature menopause and infertility due to reduced secretion of gonadotropins. In addition, low estradiol levels lead to reduced libido, vaginal dryness and dyspareunia^14^ ^,^ ^16^ ^-^ ^17^.

In the category “male sexuality”, erectile dysfunction was the main focus of the studies^19^ ^-^ ^21^ ^,^ ^23^. In two studies, the authors also investigated sexual desire and satisfaction^19^ ^-^ ^20^ and one study^22^ investigated the consequences for newborns generated by men who impregnated their partners while using drugs with mycophenolic acid.

In the category regarding both genders, the evaluation of sexual function was the main topic of the primary studies. In three studies, measurements were made using the International Index of Erectile Function (IIEF) and Female Sexual Function Index (FSFI)^3^ ^,^ ^26^ ^-^ ^27^. The authors also used other scales, such as: the Beck Depression Inventory (instrument to evaluate depression), the Short Form (36) Health Survey (scale to evaluate quality of life)^26^, the Locke-Wallace Marital Adjustment Test (instrument to evaluate marital satisfaction)^3^ and the Female Distress Scale - Revised (FSDS-R), a scale that evaluates personal suffering related to sexuality^27^. In two studies^24^ ^-^ ^25^, the authors adopted their own data collection questionnaire.

In transplanted women, the menstrual cycle returns to normal up to one year after surgery, allowing women of childbearing age to be mothers after one or two year of their transplantation, according to doctor’s recommendations^14^ ^,^ ^29^. However, transplanted women need special care due to the use of teratogenic drugs during time of pregnancy. Therefore, pregnancy must be planned in order to avoid maternal-fetal complications and transplant graft dysfunction. The results of the primary studies included in the review (n=3) showed that transplanted women were little aware of the problem and health professionals did not provide proper orientation about the need for family planning and effective contraceptive methods. Therefore, female transplant recipients exposed themselves, the fetus and the organ received to risks^16^ ^-^ ^18^.

There are gaps in knowledge about which contraceptive method is recommended for transplanted women. In some studies, the authors contraindicate the use of the IUD, due to the potential risk of infection, especially in people with immunosuppression^30^ ^-^ ^31^. In other studies, the authors argue that the use of the intrauterine device by transplanted women is safe and the greater occurrence of pelvic infection due to immunosuppression is a myth, and there is no absolute contraindication for its use^32^ ^-^ ^33^. Therefore, further studies are necessary to investigate which contraceptive methods are most suitable for this group of women. However, it is clear that the method of choice should be one that does not present drug interaction with immunosuppressants and that does not pose a risk to the health of the transplant recipient. The need to use condoms is emphasized, since, despite having a failure rate of 18%, the condom is still the only barrier method capable of preventing against sexually transmitted infections^34^.

It is necessary to conduct studies on the type of delivery most appropriate for women who were submitted to transplantation. Vaginal birth can minimize complications that could occur in a caesarean section, since women who underwent transplant surgery are likely to have adhesions and fibrosis resulting from abdominal surgery. However, because the gestation of these women is considered of high-risk, caesarean sections occur more often than vaginal delivery^35^ ^-^ ^37^. It is evident that the route of delivery selected should be one that does not cause risks to the health of the fetus and the mother.

Breastfeeding by transplant mothers is a controversial subject, and scholars must conduct future research on the care of newborns. Breastfeeding is discouraged by some authors, while others question the benefit-risk of depriving the newborn of maternal nutrition, since the fetus has already been exposed to immunosuppressive drugs during gestation. In addition, the amount of drugs excreted in breast milk would not be sufficient to cause harm to the baby^37^ ^-^ ^39^.

As a suggestion, it would be of great value to create a Brazilian registry system on pregnant women and transplant recipients, so that post-transplant maternity and paternity and the effects of drugs on fertility, pregnancy and conception could be investigated. In addition, transplantation centers in Brazil must record data regarding this problem in the Transplant Pregnancy Registry International.

Regarding the health of children generated by parents using immunosuppressive drugs, according to the Food and Drug Administration (FDA), the prevalence of health problems observed was similar to outcomes found in the general population^22^.

The guidance and orientation provided by health professionals about birth control and the use of contraceptive methods cannot be only for the female audience, since men who want to be fathers also require care during the pregnancy of the partner. This is due to the use of teratogenic immunosuppressive drugs during conception, as well as the use of some classes of anti rejection drugs that provoke testicular toxicity, affecting male fertility^22^ ^,^ ^40^.

In men, clinical signs of hypogonadism include gynecomastia, hair thinning, testicular atrophy, reduction of the prostate and oligospermia, resulting in complications such as erectile dysfunction and decreased libido and fertility. The etiology of terminal liver disease is a risk factor for the occurrence of erectile dysfunction. In alcoholic cirrhosis the chance of having this dysfunction is greater, since alcohol has toxic effects and exerts direct action on the pituitary axis, interfering in the production of hormones such as testosterone and impairing sexual health^19^ ^,^ ^21^ ^,^ ^23^.

Decreased libido and sexual interest were identified in both genders. The transplant can recover the patient’s health by replacing the diseased organ with a healthy one, and theoretically, the pathologies involved in the cause of sexual dysfunction are resolved. However, different factors are involved in the success of the transplantation and the organ replacement requires special lifelong care and may cause secondary problems. Thus, transplantation is not always capable of restoring the full sexual function of the transplanted individuals and, when there is an improvement, it is still lower than in the general population, and may take up to eighteen months to occur^19^.

In the analysis of the occurrence of sexual dysfunction in men and women who were candidates and recipients of other solid organs such as kidney and heart, the results were similar to those identified in liver transplantation, that is, not all transplanted patients presented global recovery of sexual health. The prognosis of sexual dysfunction is related to the sexual function presented by the individual before being affected by the disease that led to the transplantation. In other words, patients who had good sexual health, even with sexual function impaired by the disease, had a greater chance of recovery after transplantation than those who already had low sexual health prior to disease^41^.

The results of studies included in the review pointed out that contraceptive counseling for men and women is deficient and often occurs at an inappropriate time, that is, shortly after transplantation, when the recipient receives a high amount of information and goes through new experiences that require an adaptation phase, even if the person has been well prepared, theoretically, for the confrontations subsequent to surgery^16^ ^-^ ^18^. When the guidelines for birth control are not assimilated or incorporated because the patient is focused on recovery after transplantation, sexuality will become more important after primary basic needs are met.

The risk of contracting sexually transmitted infections is higher in the transplanted population because of the degree of immunosuppression. In the phase prior to transplantation, liver disease also depresses the patients’ immune system, favoring the development of HPV. The chances of this pathology increase when the etiology of cirrhosis is the hepatitis C virus, in both men and women, causing lesions in the cervical, uterine and anal regions. Therefore, this public should receive greater attention regarding the screening of cancer precursor lesions related to HPV infection or activation. This evaluation should be performed with greater frequency in transplant candidates and recipients, since women with immunosuppression present a higher risk of developing cervical cancer when compared to healthy women^15^ ^,^ ^42^.

As already pointed out, the majority (n=14) of the primary studies included in the review was classified with level of evidence IV (qualitative or descriptive study), indicating the need to conduct future research with strong designs on the issue of interest, providing evidence that can generate impact on decision making. No study was identified in the LILACS database, which may indicate lack of publications on the topic of interest in Latin American and Caribbean journals.

## Conclusion

The sexual alterations most prominent among candidates and recipients of liver transplant were sexual dysfunctions, manifested in men by impotence and in women by amenorrhea and dyspareunia. Decreased libido and sexual interest was also evident in this public.

Satisfactory sexual health is fundamental for quality of life and, therefore, must be part of the anamnesis. Thus, the qualification of health professionals for the acquisition of knowledge and development of skills to address this issue with patients is of great relevance, considering, above all, their difficulties in exploring the sexual health of patients. The taboo on individual and cultural experiences with sexuality, as well as lack of training since college, influences the professional performance, since sexuality is underestimated and considered less relevant than the other problems presented by the patients.

The synthesis of knowledge indicated the need to intensify efforts for the development of studies with methodologies capable of producing strong evidence, since the studies analyzed did not present robust levels of evidence. In addition, there is a scarce production of national research on sexuality in liver transplant candidates and recipients, as well as of studies that assess the long-term consequences of transplantation in the sexuality of men and women affected by chronic liver disease. Therefore, it is necessary to conduct future research on this subject. The incorporation of evidence into clinical practice can help the implementation of actions and interventions effective for patient care, according to their preferences, and support health professionals in the decision making process.
